# Comparing the Osteogenic Potentials and Bone Regeneration Capacities of Bone Marrow and Dental Pulp Mesenchymal Stem Cells in a Rabbit Calvarial Bone Defect Model

**DOI:** 10.3390/ijms20205015

**Published:** 2019-10-10

**Authors:** Yu-Chieh Lee, Ya-Hui Chan, Sung-Chih Hsieh, Wei-Zhen Lew, Sheng-Wei Feng

**Affiliations:** 1 Department of Obstetrics and Gynecology, Taipei Medical University Hospital, Taipei 110, Taiwan; d119097012@tmu.edu.tw; 2 School of Dentistry, College of Oral Medicine, Taipei Medical University, Taipei 110, Taiwan; babypg666666@gmail.com (Y.-H.C.); endo@tmu.edu.tw (S.-C.H.); b202094090@tmu.edu.tw (W.-Z.L.); 3 School of Oral Hygiene, College of Oral Medicine, Taipei Medical University, Taipei 110, Taiwan; 4 Division of Prosthodontics, Department of Dentistry, Taipei Medical University Hospital, Taipei 110, Taiwan

**Keywords:** mesenchymal stem cells, dental pulp stem cells, bone marrow stem cells, bone regeneration, calvarial defect

## Abstract

The bone regeneration efficiency of bone marrow mesenchymal stem cells (BMSCs) and dental pulp mesenchymal stem cells (DPSCs) combined with xenografts in the craniofacial region remains unclear. Accordingly, this study commenced by comparing the cell morphology, cell proliferation, trilineage differentiation, mineral synthesis, and osteogenic gene expression of BMSCs and DPSCs in vitro. Four experimental groups (empty control, Bio-Oss only, Bio-Oss+BMSCs, and Bio-Oss+DPSCs) were then designed and implanted in rabbit calvarial defects. The BMSCs and DPSCs showed a similar morphology, proliferative ability, surface marker profile, and trilineage-differentiation potential in vitro. However, the BMSCs exhibited a higher mineral deposition and expression levels of osteogenic marker genes, including alkaline phosphatase (ALP), runt related transcription factor 2 (RUNX2), and osteocalcin (OCN). In the in vivo studies, the bone volume density in both MSC groups was significantly greater than that in the empty control or Bio-Oss only group. Moreover, the new bone formation and Collagen I / osteoprotegerin protein expressions of the scaffold+MSC groups were higher than those of the Bio-Oss only group. Finally, the Bio-Oss+BMSC and Bio-Oss+DPSC groups had a similar bone mineral density, new bone formation, and osteogenesis-related protein expression. Overall, the DPSCs seeded on Bio-Oss matched the bone regeneration efficacy of BMSCs in vivo and hence appear to be a promising strategy for craniofacial defect repair in future clinical applications.

## 1. Introduction

Craniofacial bone defects occur for many reasons, such as bone disease, trauma, severe infection, congenital malformations, and tumor resections, and frequently require bone tissue reconstruction for medical or aesthetic reasons [1]. Tissue engineering and regenerative medicine offer novel treatment modalities for such defects, including bone augmentation procedures by using scaffolds, stem cells, and cell signaling [2]. However, while autogenous bone grafts are the gold standard for bone regeneration, donor site morbidity and the limited availability of bone volume restrict their practical application in clinical contexts [3,4]. Consequently, various artificial bone grafting materials, such as xenografts and synthetic grafts, have been developed as substitutes for autogenous bone grafts [3]. However, current commercially available materials have only a poor osteogenic and osteoinductive ability [5,6]. In view of this, cell-based bone tissue engineering, including the use of osteoinductive stem cells combined with an osteoconductive scaffold, has recently attracted growing attention as an alternative treatment modality for the repair of bone defects [7,8].

Mesenchymal stem cells (MSCs) are multipotent stromal cells with the ability to undergo self-renewal and multi-lineage differentiation [9,10,11,12]. MSCs can additionally exert a biologically supportive function through paracrine actions and trophic factors [13,14]. Bone marrow mesenchymal stem cells (BMSCs) are readily available and possess a high osteogenic capacity. As a result, they are considered to be one of the most appropriate stem cell populations for bone regeneration and are thus widely used for efficiency comparison purposes with other cell sources [15,16,17,18]. Several studies have demonstrated that BMSCs combined with suitable scaffolds are more effective in promoting new bone formation than scaffolds alone in animal bone defect models [15,18]. Moreover, immunofluorescence staining results have shown that after the addition of BMSCs, the newly-formed bone expresses significantly higher bone differentiation markers (e.g., ALP (alkaline phosphatase), RUNX2 (runt related transcription factor 2), and OCN (osteocalcin)) [16,19]. However, BMSCs have several practical drawbacks for clinical applications, including painful collection methods, donor site damage, and lower proliferation/differentiation capacities with advancing donor age [11,20].

Accordingly, dental pulp mesenchymal stem cells (DPSCs) have attracted growing attention as an alternative cell source. DPSCs have many advantages for bone regeneration, including a high proliferation rate, a good osteogenic differentiation potential, and favorable paracrine and immunomodulatory properties [9,21,22,23]. Furthermore, the ease of isolation and accessibility of DPSCs from removed and discarded teeth offers an abundant source of cells for regenerative medicine with minimal risk of complications [24,25]. Both in vivo and in vitro studies have confirmed the feasibility of DPSCs for osteogenesis and bone regeneration [6,26,27]. Moreover, several studies have shown that the combination of DPSCs and biomaterials provides an effective protocol for bone defect reconstruction and craniofacial bone regeneration [20,22,28,29,30]. 

Although BMSCs and DPSCs are both promising cell sources for bone regeneration, most studies have shown that BMSCs have a higher osteogenic ability than DPSCs [31,32,33]. However, comparative studies on the osteogenic potentials of BMSCs and DPSCs in vitro and bone regeneration in vivo have yielded conflicting results. In particular, some studies have reported that DPSCs perform better than BMSCs [6,34,35], while others have shown no obvious difference between them [36]. The apparent inconsistency in the reported results arises since most studies focus only on in vitro analyses [34,35,37,38] or compare the bone regeneration capacities of the two cells only in small animal models [34,36]. Furthermore, the osteogenic effects of stem cells may reveal different regenerative potentials when implanted in different anatomic regions or in different animal models [23]. As a result, it is still unclear which cell type (i.e., BMSC or DMSC) should be recommended for clinical translation in craniofacial bone regeneration.

Accordingly, the present study commences by comparing BMSCs and DMSCs in vitro with regard to their proliferation, differentiation, mineralization, and osteogenic gene expression. In vitro studies are then performed to investigate whether the combination of Bio-Oss scaffold with BMSCs and DMSCs promotes improved bone regeneration and osteogenesis-related protein expression in a rabbit calvarial defect model compared to an empty control group or Bio-Oss scaffold-only group.

## 2. Results

### 2.1. Characterization and Proliferation of BMSCs and DPSCs

**Figure 1:**
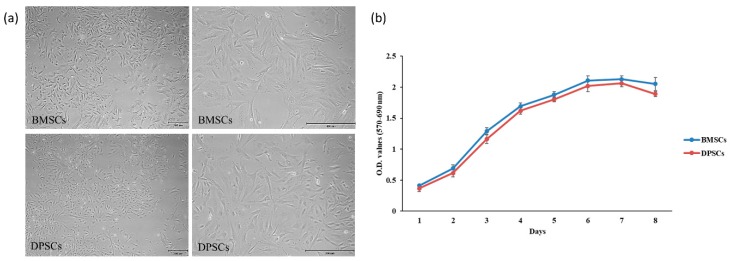
(**a**) Images of BMSCs and DPSCs at passage 3 after 3 days of culture. Images of BMSCs and DPSCs at × 10 and × 40 magnification revealed that both cell types exhibited a healthy fibroblastic morphology typical of MSCs. Scale bars = 200 μm. (**b**) Proliferation rates of BMSCs and DPSCs. A similar proliferation rate and tendency was found for the two cell types.

The BMSCs and DPSCs both showed a typical spindle-shaped fibroblast-like morphology with a homogeneous shape and size (Figure 1a). Furthermore, as shown in Figure 1b, both cell types exhibited a similar proliferative ability; with a gradual increase in the cell number over time. Although the proliferative ability of the BMSCs was slightly higher than that of the DPSCs, no statistically significant difference was found between them. 

### 2.2. Fluorescence-Activated Cell Sorting Analysis (FACS) of BMSCs and DPSCs

**Figure 2:**
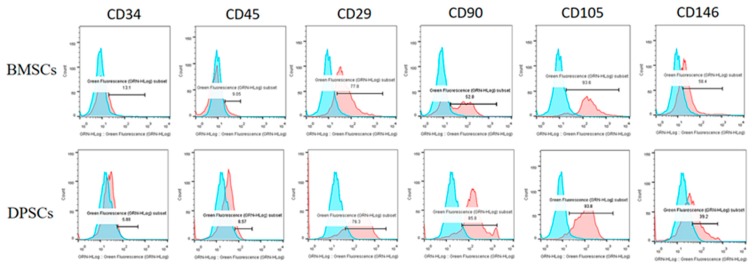
Characterization of surface marker profiles for BMSCs and DPSCs. The flow cytometry results revealed that the BMSCs and DPSCs were positive for CD29, CD90, CD105, and CD146, but negative for CD34 and CD45.

The surface antigens of the BMSCs and DPSCs were analyzed using flow cytometry (Guava EasyCyte Mini Base System, Guava Technologies, Millipore, Hayward, CA, USA). The data were analyzed using FlowJo software (TreeStar Inc., Ashland, OR, USA). The results revealed that the two cell types were positive for CD29, CD90, CD105, and CD146, but negative for CD34 and CD45 (Figure 2). 

### 2.3. Trilineage Differentiation Capacities of BMSCs and DPSCs

**Figure 3:**
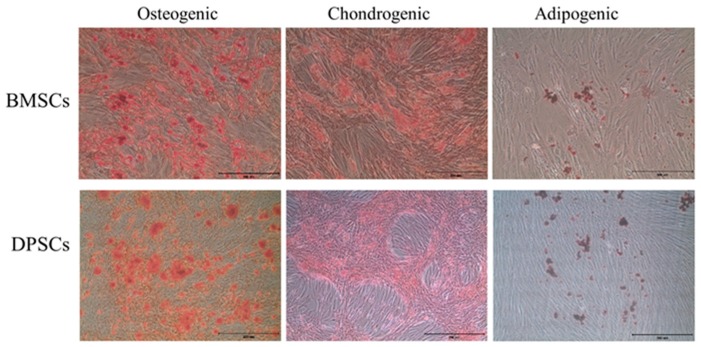
Images showing in vitro trilineage differentiation of cultured BMSCs and DPSCs. The trilineage differentiation capacities of the BMSCs and DPSCs were confirmed by alizarin red for extracellular calcium, safranin O for extracellular cartilage, and oil red O for intracellular lipid accumulation. Scale bars = 100 μm.

The osteogenic, chondrogenic and adipogenic differentiation potentials of the BMSCs and DPSCs were evaluated with different induction media in vitro. The BMSCs exhibited a significantly higher mineral matrix deposition than the DPSCs after 14 days of osteogenic induction (Figure 3). However, both cell types exhibited a similar robust glycosaminoglycan deposition upon chondrogenic induction. Finally, lipid droplets were observed in both cell types upon adipogenic induction. No significant difference was found between the BMSCs and DPSCs as regards their chondrogenic and adipogenic differentiation potentials. 

### 2.4. Osteogenesis and Gene Expression of BMSCs and DPSCs

**Figure 4:**
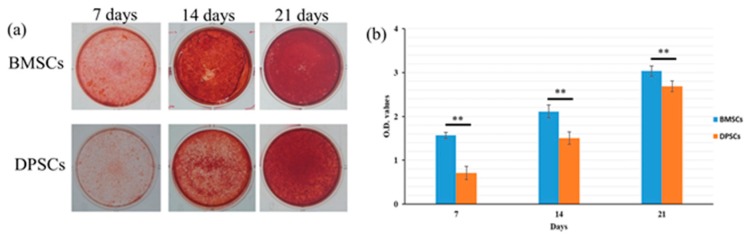
Comparison of osteogenic differentiation capacities of BMSCs and DPSCs. (**a**) Mineral depositions of BMSCs and DPSCs were detected by alizarin red staining following 7, 14, and 21 days of osteogenic induction. (**b**) The BMSCs showed a higher osteogenic differentiation ability than the DPSCs with statistically significant differences at different time points (** *p* < 0.01).

**Figure 5:**
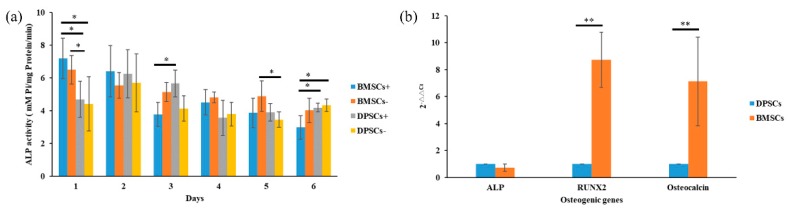
Comparison of ALP (alkaline phosphatase) activity and osteogenesis-related gene expression of BMSCs and DPSCs. (**a**) The expression of ALP activity of the BMSCs and DPSCs in differentiation media and regular culture medium was evaluated after 1, 2, 3, 4, 5 and 6 days of culture. (**b**) The relative osteogenic lineage gene expression levels (ALP, RUNX2 (runt related transcription factor 2), and Osteocalcin) of the BMSCs and DPSCs were assessed by qRT-PCR (* *p* < 0.05, ** *p* < 0.01).

Alizarin red staining was used to quantify the mineral matrix depositions of the BMSCs and DPSCs after 7, 14, and 21 days of osteogenic induction culture (Figure 4a). The mineral matrix deposition of the BMSCs was found to be significantly higher than that of the DPSCs after 7 and 14 days. However, after 21 days of culture, no obvious difference was observed between the two cell types (Figure 4b). The alkaline phosphatase (ALP) activity of the BMSCs was significantly higher than that of the DPSCs both with and without osteogenic induction; particularly after 1 day of culture (Figure 5a). After 3 days of culture, the DPSCs with osteogenic induction revealed significantly more ALP activity than the other groups. However, no significant differences in the ALP activity were observed among the four groups after 2 and 4 days of culture In other words, the BMSCs displayed a stronger ALP activity and in vitro calcium deposition ability than the DPSCs during the early differentiation period, but exhibited a similar osteogenic potential during the late differentiation period. 

The osteogenic gene expressions of RUNX2 (runt related transcription factor 2) and OCN (osteocalcin) were markedly higher for the BMSCs than for the DPSCs after 24 hours of culture (Figure 5b). However, a similar gene expression of ALP was found for both cell types. 

### 2.5. Micro-CT Measurements

**Figure 6:**
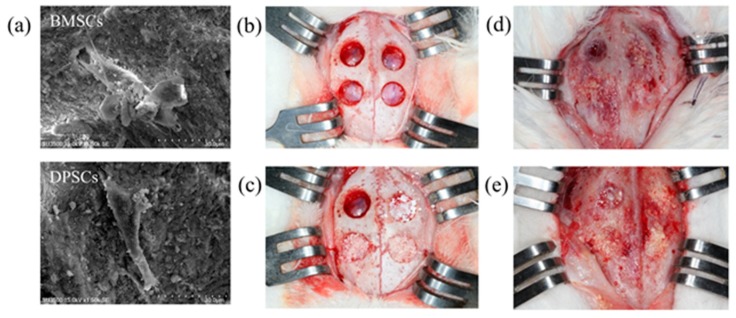
Rabbit calvarial bone defects and microscopic analysis. (**a**) Scanning electron microscopy (SEM) images of BMSCs and DPSCs cultured on Bio-Oss scaffolds. (**b**) Rabbit calvarial bone defects. (**c**) Local transplantation of MSCs combined with Bio-Oss scaffolds. (**d**) Macroscopic view of regenerative areas after 3 weeks of healing. (**e**) Macroscopic view of regenerative areas after 6 weeks of healing.

**Figure 7:**
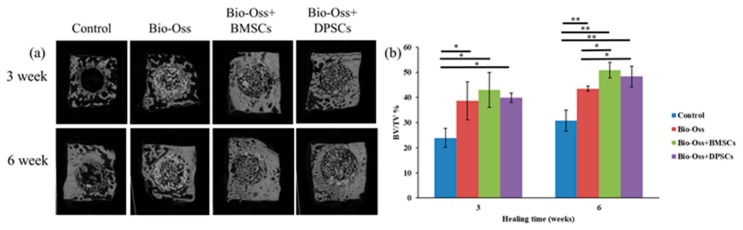
(**a**) Micro-CT images of defect regions in control, Bio-Oss, Bio-Oss+BMSC, and Bio-Oss+DPSC groups at 3 and 6 weeks. Comparison of BV/TV values among the four experimental groups at (**b**) 3 and (**c**) 6 weeks after surgery. The results show significant differences in the bone formation among the various groups (* *p* < 0.05, ** *p* < 0.01).

The osteogenic effects of the BMSCs and DPSCs on bone defect repair were investigated using a rabbit calvarial bone critical defect model. A total of 30 mg of Bio-Oss bone grafting material containing 1 × 10^6^ BMSCs or DPSCs was implanted into 6 mm defect cavities prepared using a trephine bure (Figure 6). Tissue samples were collected for micro-CT, histological, and immunohistochemical evaluations after 3 and 6 weeks of healing (Figure 6d,e). As shown in Figure 7a, treatment of the calvarial defects with undifferentiated BMSCs or DPSCs significantly improved the rate of bone defect bridging and the amount of newly-formed bone compared to the control group or scaffold-only group. The 3D-reconstructed micro-CT images of the bone defects revealed a fusion of the new bone with the host bone and the formation of mineralized interconnections between the Bio-Oss particles in the Bio-Oss only, Bio-Oss+BMSC, and Bio-Oss+DPSC groups. Furthermore, the Bio-Oss+BMSC and Bio-Oss+DPSC groups showed a larger amount of bony bridges than the Bio-Oss group and a smaller amount of Bio-Oss grafting material particles.

The BV/TV (bone volume/tissue volume) ratios in the defect areas of the MSC-treated groups were significantly different (*p* < 0.05) than those in the non-MSC-treated groups at both 3 and 6 weeks after surgery (Figure 7b). In particular, after 3 weeks, the BV/TV ratios in the Bio-Oss only, Bio-Oss+BMSC, and Bio-Oss+DPSC groups were 38.6 ± 7.5%, 42.9 ± 1.8% and 39.9 ± 6.9%, respectively, higher than that in the empty control group (23.8 ± 3.7%). Notably, while the Bio-Oss+BMSC group showed a higher BV/TV value than the Bio-Oss only or Bio-Oss+DPSC group, no significant difference was found among the three groups. After 6 weeks, the BV/TV ratios in the Bio-Oss+BMSC and Bio-Oss+DPSC groups were 50.8 ± 4.0% and 48.3 ± 3.0%, respectively, and were both significantly higher than that of the empty control group (30.7 ± 4.1%) or Bio-Oss only group (43.5 ± 0.9%). 

### 2.6. Histological Observations and Histomorphometric Analysis

**Figure 8:**
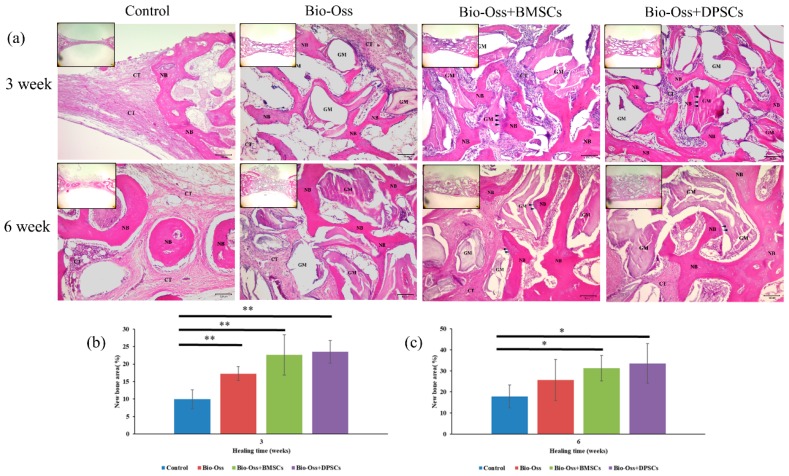
(**a**) Hematoxylin and eosin (H&E) stained images of bone defect regions at 3 and 6 weeks in control, Bio-Oss, Bio-Oss+BMSC, and Bio-Oss+DPSC groups. The black arrowheads indicate osteoblasts at the interface between the bone grafting materials and the newly-formed bone. New bone area percentages in defect region at (**b**) 3 weeks and (**c**) 6 weeks after surgery, as assessed by histomorphometric analyses. (NB = new bone; GM = graft material; CT = connective tissue; H&E = hematoxylin and eosin. Scale bars = 100 m.) (* *p* < 0.05, ** *p* < 0.01.).

None of the bone defect specimens showed any signs of inflammation or infection induced by the Bio-Oss grafting material or mesenchymal stem cells. The histological and histomorphometric results revealed a higher new bone formation in the Bio-Oss+BMSC and Bio-Oss+DPSC groups than in the empty control and Bio-Oss only groups (Figure 8). Figure 8a shows representative histological sections of the four experimental groups at 3 and 6 weeks after surgery. For the empty control group, the bone defect is filled mainly with connective tissue after 3 weeks. In other words, no bony tissue is observed and only fibrous scar tissue formation is evident. Furthermore, after 6 weeks of healing, only mild new bone formation is observed at the defect margin. For the Bio-Oss only group, moderate new bone formation is seen around the grafting material after just 3 weeks. Furthermore, the grafting material is resorbed and replaced by new bone after 6 weeks. The Bio-Oss+BMSC and Bio-Oss+DPSC groups both show a higher new bone formation in the bone defect area than the empty control group or Bio-Oss only group. In addition, both groups not only induce horizontal bone defect closure, but also maintain the new bone height. After 3 weeks, direct contact is observed between the new bone and the Bio-Oss grafting material. In other words, osteoconductivity occurs at the lateral defect margin. Moreover, osteocytes are seen in the newly-formed thickened bone in both the Bio-Oss+BMSC group and the Bio-Oss+DPSC group. In addition, considerable numbers of osteoblastic cells can be seen on the surface of the newly-formed bone and at the interface between the bone graft and the new bone in the Bio-Oss+BMSC and Bio-Oss+DPSC groups. In general, the results indicate that the osteoconductive capacity of Bio-Oss scaffold is improved by the addition of BMSCs or DPSCs.

The amount of newly-formed bone in the Bio-Oss only (17.2 ± 1.9%), Bio-Oss+BMSC (22.6 ± 3.2%), and Bio-Oss+DPSC (23.4 ± 5.7%) groups was significantly higher than that in the control group (9.9 ± 2.6%) at 3 weeks after surgery (Figure 8b). No statistically significant difference was observed between the amounts of newly-formed bone in the Bio-Oss+BMSC and Bio-Oss+DPSC groups. However, both groups showed a greater amount of newly-formed bone than the Bio-Oss only group. The amount of newly-formed bone increased in all of the experimental groups after 6 weeks (Figure 8c). The bone formation in the Bio-Oss+BMSC (31.2 ± 6.0%) and Bio-Oss+DPSC (33.5 ± 9.3%) groups was significantly higher than that in the control group (17.8 ± 5.4%). However, no significant difference was observed between the Bio-Oss only group (25.6 ± 9.7%) and the control group.

### 2.7. Immunohistochemical Evaluations

**Figure 9:**
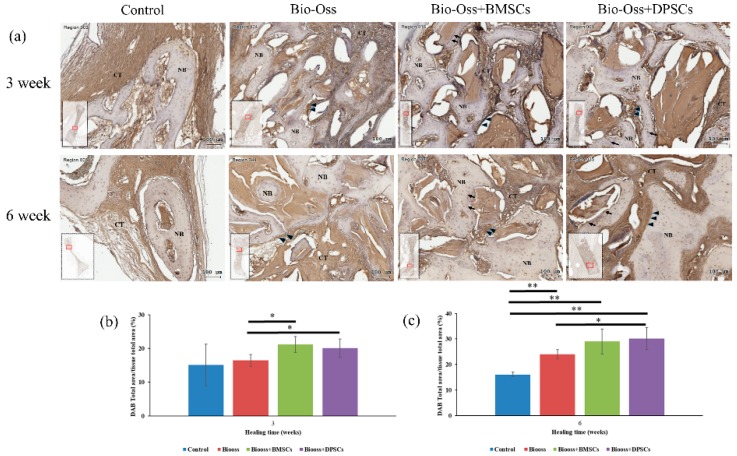
(**a**) Images of Collagen I immunostaining in bone defects in control, Bio-Oss, Bio-Oss+BMSC, and Bio-Oss+DPSC groups at 3 and 6 weeks. The black arrowheads indicate osteoblasts at the interface between the bone grafting materials and the newly-formed bone. Collagen I immunostaining percentages in defect region at (**b**) 3 weeks and (**c**) 6 weeks after surgery as assessed by DAB total area/tissue total area analyses. (NB = new bone; GM = graft material; CT = connective tissue. Scale bars = 100 μm.) (* *p* < 0.05, ** *p* < 0.01.).

**Figure 10:**
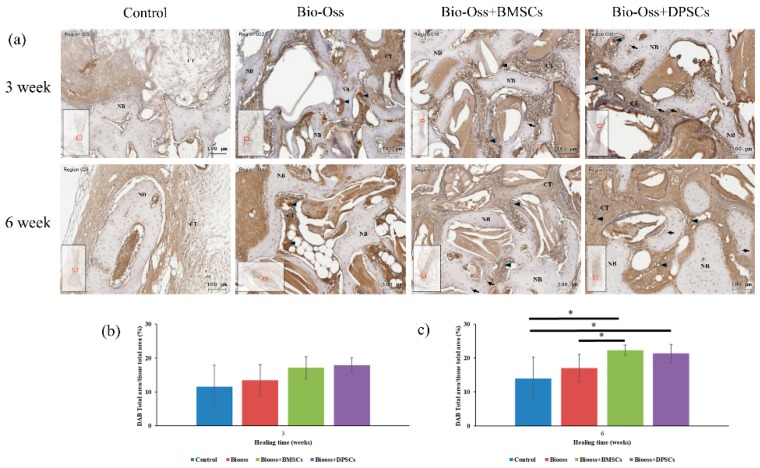
(**a**) Images of osteoprotegerin (OPG) immunostaining in bone defects in control, Bio-Oss, Bio-Oss+BMSC, and Bio-Oss+DPSC groups at 3 and 6 weeks. The black arrowheads indicate osteoblasts at the interface between the bone grafting materials and the newly-formed bone. OPG immunostaining percentages in defect region at (**b**) 3 weeks and (**c**) 6 weeks after surgery as assessed by DAB total area/tissue total area analyses. (NB = new bone; CT = connective tissue. Scale bars = 100 μm.) (* *p* < 0.05).

The Collagen I and OPG-positive regions in the defect area were significantly more pronounced in the Bio-Oss+BMSC and Bio-Oss+DPSC groups than in the Bio-Oss only and empty control groups (Figure 9 and Figure 10). For the control group, the osteoblasts, osteocytes, and connective tissue matrix stained only weakly for Collagen I and OPG. The Collagen I and OPG immunohistochemistry results showed positive staining for some osteocyte, osteoblasts, and connective tissue matrix in the Bio-Oss only, Bio-Oss+BMSCs and Bio-Oss+DPSCs groups. A strong Collagen I and OPG staining of the osteoblasts (arrow), osteocytes (arrowheads), and connective tissue matrix was observed in the Bio-Oss+BMSCs and Bio-Oss+DPSCs groups. In addition, osteoblasts (arrowheads) and osteocytes (arrows) at the periphery of the new bone were also positive for Collagen I and OPG. 

The immunostaining percentages of Collagen I and OPG in the four experimental groups at 3 and 6 weeks were assessed by means of 3,3′-diaminobenzidine tetrahydrochloride (DAB) total area/tissue total area analyses. The amount of Collagen I staining was significantly higher in the Bio-Oss+BMSC (21.1 ± 2.3%) and Bio-Oss+DPSC (20.0 ± 2.6%) groups than in the Bio-Oss only group (16.4 ± 1.7%) at week 3 (Figure 9b). Furthermore, the amount of Collagen I staining was significantly higher in the Bio-Oss only (24.0 ± 1.8%), Bio-Oss+BMSC (29.0 ± 4.9%), and Bio-Oss+DPSC (30.1 ± 4.3%) groups than in the empty control group (16.0 ± 0.9%) at week 6 (Figure 9c). Furthermore, a significant difference was observed between the Bio-Oss only group and the Bio-Oss+DPSC group. For the OPG staining, no significant differences were found among the empty control group (11.5 ± 6.3%), the Bio-Oss only group (13.4 ± 4.5%), the Bio-Oss+BMSC group (17.1 ± 3.2%), and the Bio-Oss+DPSC group (17.9 ± 2.2%) at week 3 (Figure 10b). However, the amount of OPG staining was significant higher in the Bio-Oss+BMSC (22.3 ± 1.5%) and Bio-Oss+DPSC (21.3 ± 2.6%) groups than in the empty control group (13.9 ± 6.2%) or Bio-Oss only group (17.0 ± 4.0%) at week 6 (Figure 10c). Furthermore, a significant difference was found between the Bio-Oss only group and the Bio-Oss+BMSC group.

## 3. Discussion

To the best of the authors’ knowledge, the present study is the first reported attempt in the literature to comprehensively evaluate the proliferation and osteogenic differentiation abilities of BMSCs and DPSCs in vitro and to compare their new bone formation efficiency after 3 and 6 weeks of healing in a large-animal (rabbit) calvarial bone defect model. Overall, the in vitro results have shown that the BMSCs possess a higher osteogenic differentiation potential than the DPSCs. However, the two cell types exhibit a similar morphology, proliferative ability, surface marker expression, and trilineage differentiation. Furthermore, the in vivo results have shown that the bone regenerative capacity of the DPSCs is similar to that of the BMSCs. 

Craniofacial bone defects caused by cancer surgery, trauma, pathologies or congenital malformation, are major health problems and pose particular challenges for bone reconstruction [1]. Apart from standard treatment modalities using bone grafting materials, stem cell-based therapies also represent a promising new approach for bone repair and regeneration. Mesenchymal stem cells (MSCs) share several common characteristics in vitro, including a fibroblast-like morphology, a good proliferation capacity, multilineage differentiation, and an immunomodulatory function [7]. Furthermore, MSCs combined with biological scaffolds appear to represent an ideal strategy for regenerative medicine [15,22]. However, conflicting results have been reported in the literature for BMSCs and DPSCs, which leads to ambiguity regarding the practical usefulness of DPSCs [6,31,33,38]. Furthermore, the question as to which tissue source is optimal for repairing craniofacial defects remains unanswered. 

In the present study, the cell morphology, proliferation, surface marker profiles, trilineage differentiation, mineral synthesis, and osteogenic gene expression of BMSCs and DPSCs were compared by means of in vitro assays. The findings revealed that both MSCs exhibit the typical morphology, proliferation rate, surface markers, and trilineage differentiation properties of MSCs. Similar results were also reported in a porcine model [6]. However, other studies have indicated that DPSCs have a higher proliferation rate, availability, and cell number than BMSCs [21,38,39]. This discrepancy suggests that MSCs isolated from different tissue sources and individuals may exhibit a different cell proliferation rate. The present results have additionally shown that the osteogenic potential of BMSCs is better than that of DPSCs (as confirmed by the calcium deposition ability, ALP activity, and gene expression analysis results). This finding is consistent with that of most previous studies, which also showed that the osteogenic differentiation potential of BMSCs is greater than that of DPSCs [33,38,39]. However, compared with BMSCs, DPSCs can survive in vitro for a longer time, exhibit a higher growth rate, and produce an extracellular matrix with less immunosuppressive activity [6,20,25,40]. 

In tissue engineering, scaffolds can influence the cellular response and support bone formation, but must be specific to the regenerated tissue [23]. Hence, in choosing and designing an ideal scaffold material, biocompatibility, biodegradability, and superior mechanical properties with no adverse side effects are the main concerns [21]. Many biomaterials have been used as carriers for loading MSCs in bone tissue engineering, including collagen sponge, hydroxyapatite (HA)/β-tricalcium phosphate (β-TCP), demineralized bone matrix (DBM), and Bio-Oss [16,18,22,28]. Among these materials, xenografts and synthetic bone graft materials are the most commonly used in clinical settings due to their safety and efficacy. However, these materials show only a limited osteoconduction capacity for bone regeneration. 

In the present study, Bio-Oss scaffold was specifically chosen as the carrier for the MSCs since it is the most widely used and reliable biomaterial among all materials currently used in dentistry for the augmentation of bony defects [16]. The histological findings (Figure 8) confirmed that Bio-Oss has good biocompatibility and osteoconductivity, and therefore facilitates the attachment of bone-forming cells, supports the formation of new bone, and maintains the original defect space. Moreover, a large amount of newly-formed bone and a considerable number of osteoblastic cells lining the bone surface and interface between the new bone and the scaffold were also observed. These findings suggest that the osteoinduction characteristics of Bio-OSS are aided by the addition of BMSCs and DPSCs. This inference is supported by the strong Collagen I staining results, which indicate the presence of calcium-phosphate rich mineral in the extracellular matrix and the osteoblast-associated OPG staining within and at the periphery of the healing defects (Figure 9 and Figure 10). Studies with similar conditions were previously published by Yamada et al. for alveolar ridge bone defects in a canine model and by Nakajima et al. in calvarial defects of immunodeficient mice [36,41]. In general, the results presented in these studies indicated that biomaterials combined with BMSCs or DPSCs have an equal ability to promote bone regeneration and implant osseointegration. However, another study reported that the osteogenic ability of DPSCs was superior to that of BMSCs when combined with polycaprolactone-hyaluronic-tricalcium phosphate scaffold in a porcine calvarial model [6]. In other words, although DPSCs show an inferior osteogenic potential to BMSCs in in vitro assays, scaffolds combined with DPSCs achieve a regeneration outcome similar to that of BMSCs in in vivo models.

The possible mechanisms responsible for promoting in vivo tissue regeneration in damaged areas through the implantation of MSCs include the rescue of damaged cells, the induction of endogenous cell differentiation, the recruitment of osteogenic cells, and a modulation of the immune response [42,43]. Moreover, the MSC-secreted bioactive trophic factors comprising soluble molecule enzymes, growth factors, and interleukins, which also accelerate self-renewal, stimulate angiogenesis, and minimize inflammation [14,42,44]. These findings imply that BMSCs and DPSCs do not only participate in bone regeneration, but also modulate bone regeneration through biologically paracrine functions. However, in MSC-based therapy, the embryonic properties between the cell source and the repaired tissue should also be considered. For example, the high plasticity and multi-potential capacity of DPSCs to differentiate into several different tissues can be explained by their neural crest origin, which supports their application not only in the oral region, but also to other regions, such as craniomaxillofacial bone [23]. 

Wisdom teeth extraction is one of the most common procedures in oral surgery. Hence, harvested DPSCs can be considered as a relatively easy and powerful source of MSCs [20]. According to the results obtained in the present study, DPSCs and BMSCs combined with xenografts revealed comparable bone regeneration capacities for repairing craniofacial bone defects. However, further studies are needed to fully evaluate the mechanisms of scaffold-loaded MSCs and to determine whether such a stem cell-based approach can be successfully translated into clinical practice.

## 4. Materials and Methods

### 4.1. Animals and Ethics

Twelve adult male New Zealand white rabbits weighing between 3.5 and 4.0 kg were used in this study for MSC culture and calvarial defect assays. The animals were individually housed in the Central Animal Facility at Taipei Medical University under a controlled climate (temperature 22 ± 2 °C, humidity 30–60 ± 5%, 12/12 h light/dark cycle) and free access to standard diet and drinking water. All of the experimental procedures were approved by the Institutional Animal Care and Use Committee of Taipei Medical University, Taipei, Taiwan (IACUC Approval No. LAC-2017-0126, 6 May 2017) under the ARRIVE guidelines [45]. 

### 4.2. Isolation and Culture of BMSCs and DPSCs

Approximately 1 mL of bone marrow was harvested via needle aspiration from the tibia and femoral bones of the anesthetized rabbits and suspended in 2 mL of phosphate-buffered saline (PBS) solution [46]. The bone marrow suspension was layered on 3 mL Ficoll-Hypaque-Plus solution (GE Healthcare BioSciencesCorp., Piscataway, NJ, USA) for density gradient centrifugation at 400 *g* for 30 min in a 15 mL centrifuge tube. The resulting mononuclear cell layer was collected and suspended with α-minimal essential medium (α-MEM) supplemented with 15% fetal bovine serum, 1% antibiotic-antimycotic (Gibco Invitrogen, Carlsbad, CA, USA), and 100 μmol/L of L-ascorbic acid 2-phosphate (Sigma-Aldrich, St. Louis, MO, USA). The cell suspension was filtered through a 70 μm strainer to remove any cell clumps and was then plated in culture medium. The medium was changed at day 2 to remove the non-adherent cells and was then replaced every 3 days thereafter. 

Six incisors were extracted from three rabbits and the pulp tissues were carefully removed. The tissues were washed with PBS three times, minced into pieces, and prepared using a similar technique to that described for human dental pulp tissue in Reference [25]. Small pieces of pulp tissue were cultured in 3.5 cm diameter Petri dishes in complete α-minimal essential medium (α-MEM) at 37 °C in a 5% CO_2_ environment. Until 80% confluence was attained, the cells were isolated by filtering through a 70 μm strainer.

The BMSCs and DPSCs were passaged by detachment with 0.5% trypsin-EDTA solution when the respective cultures reached ≥ 70% confluence. The cells obtained at passages 2–8 were used for the in vitro experiments. 

### 4.3. Flow Cytometric Assays

The characteristic cell surface markers for each MSC were evaluated by flow cytometry (Guava EasyCyte Mini Base System, Guava Technologies, Millipore, Hayward, CA, USA). BMSCs and DPSCs were detached with Trypsin-EDTA solution, centrifuged at 1000 rpm for 10 min and fixed with 75% ethanol at −20 °C overnight. The BMSCs and DPSCs were then incubated with the following fluorescent-conjugated antibodies: CD29-FITC, CD34-FITC, CD45-FITC, CD90-FITC, CD105-FITC, and CD146-FITC (BD Biosciences) in PBS for 30 min at 4 °C in the dark. For all of the markers, immunoglobulin-1(IgG1) was used as the isotype control. The cell suspensions were analyzed using a FACSCalibur flow cytometer (Guava EasyCyte Mini Base System, Guava Technologies, Millipore, Hayward, CA, USA). The collected data were further analyzed using FlowJo software (TreeStar Inc., Ashland, OR, USA). 

### 4.4. Osteogenic, Chondrogenic, and Adipogenic Differentiation in Vitro 

For osteogenic differentiation, BMSCs and DPSCs were cultured in osteogenic induction medium (culture medium supplemented with 0.01 μM dexamethasone and 1.8 mM KH_2_PO_4_) and exchanged every 3 days for 14 days. To visualize the mineralized deposits, the cells were fixed with 4% paraformaldehyde and then stained with 2% alizarin red S solution (Sigma–Aldrich, St. Louis, MO, USA) for 10 min at room temperature.

For chondrogenic differentiation, BMSCs and DPSCs were cultured in chondrogenic induction medium (low-glucose DMEM supplemented with 1 mM sodium pyruvate, 0.1 mM dexamethasone, 10 ng/mL TGF-β, 1× insulin-transferrin-selenium-X, and 0.1 mM L-ascorbic acid 2-phosphate) for 14 days. After fixation by 4% paraformaldehyde, the cells were rinsed with 250 μL of 1% acetic acid and stained with 1% safranin O (Sigma–Aldrich, St. Louis, MO, USA) for 5 min at room temperature.

For adipogenic differentiation, BMSCs and DPSCs were cultured in adipogenic induction medium (culture medium supplemented with 10 μg/ml of insulin, 0.5 μM hydrocortisone, 500 μM 3-isobutyl-1-methylxanthine, and 60 μM indomethacin) for 21 days. After fixation by 4% paraformaldehyde, the cells were washed with 60% isopropanol and then stained with Oil Red O working solution (Sigma–Aldrich, St. Louis, MO, USA) for 10 min at room temperature. After staining, the cells were washed and observed under a bright-field illumination microscope (Eclipse TS100; Nikon Corporation, Tokyo, Japan). The observed images were captured by a CMOS camera with SPOT Advance imaging software (SPOT Idea™, Diagnostic Instruments, Inc., Sterling Heights, MI, USA).

### 4.5. Cell Proliferation Assay

The proliferative ability of the BMSCs and DPSCs was evaluated by MTT assays. Briefly, the cells were seeded in 24-well plates with a density of 1 × 10^4^ cells per well and allowed to adhere for 24 h. After incubation for 1, 2, 3, 4, 5, 6, 7, and 8 days, 50 μl of a MTT solution consisting of 0.5 mg/ml MTT (3-(4,5-dimenthylthiazol-2-yl)-2,5-diphenyltetrasoliumbromide) (Roche Applied Science, Mannheim, Germany) was added to each well. The cells were incubated for a further 4 h at 37 °C to prompt the conversion of the MTT salt to water-insoluble formazan by the viable cells. The precipitated insoluble formazan was dissolved by 500 μl DMSO. The light absorbance of the formazan concentration was then measured at a wavelength of 570 nm using a microplate reader (EZ read 2000, Biochrom Ltd, Cambridge, UK) and a reference filter with a wavelength of 690 nm. 

### 4.6. Alkaline Phosphatase (ALP) Activity Assay

The ALP activity of the BMSCs and DPSCs was determined by measuring the rate of conversion of *p*-nitrophenyl phosphate (*p*-NPP) to *p*-nitrophenol (*p*-NP) at a pH of 10.2 and temperature of 37 °C. The cells were seeded in 24-well plates at a density of 1.0 × 10^4^ cells/ml in normal culture and osteogenic medium. After incubation for 1, 2, 3, 4, 5, and 6 days, the cells were washed with PBS and lysed with 300 μl CelLyticTMP Cell Lysis Reagent (Sigma–Aldrich Co., St. Louis, MO, USA). The cell lysate was mixed and incubated with ALP assay reagent at 37 °C for 30 min and the absorbance of the resulting ALP activity was quantified by calculating the optical density (OD) values at 405 nm. The total protein content of each cell lysate was measured using a Bio-Rad protein assay kit (Bio-Rad, Hercules, CA, USA). For each cell type, the ALP enzymatic activity was expressed as micromoles of *p*-nitrophenol produced per milligram of protein per minute. 

### 4.7. Calcium Deposition Assay

After 7, 14, and 21 days of culture in osteogenic medium, the calcium depositions in the extracellular matrixes of the BMSCs and DPSCs were stained with alizarin red S (Sigma–Aldrich, St. Louis, MO, USA). Briefly, the cells were fixed with 4% paraformaldehyde, stained with 2% alizarin red S solution (Sigma–Aldrich, St. Louis, MO, USA) for 10 min at room temperature, and then washed with PBS five times and recorded as digital photographs. In performing the quantitative assay, the stained wells were eluted for 30 min with 10% cetylpyridinium chloride solution and the assay results were expressed in terms of the absorbance OD value at 590 nm. 

### 4.8. Quantitative Reverse-Transcription Polymerase Chain Reaction (qRT-PCR) Analysis

The osteogenic gene expressions of the BMSCs and DMSCs were analyzed by means of qRT-PCR. Total ribonucleic acid (RNA) was isolated using a Total RNA Mini Kit (NovelGene Biotech, Taipei, Taiwan) after 24 h of culture according to the manufacturer’s protocol. Complementary deoxyribose nucleic acid (cDNA) was then synthesized from 1 μg of the total RNA using a high-capacity cDNA Reverse Transcription Kit (Applied Biosystems^TM^, Foster City, CA, USA). The cDNA was amplified using a real-time DNA thermal analyzer (Rotor-gene 6000, Corbett Life Science, Sydney, Australia) with FastStart Universal SYBR Green Master (Roche Applied Science, Mannheim, Germany). The relative expressions of runt related transcription factor 2 (RUNX2) genes, alkaline phosphatase (ALP), and osteocalcin (OCN) were calculated using the comparative ΔC_T_ method using glyceraldehyde 3-phosphate dehydrogenase (GAPDH) for normalization purposes. The relative expression levels of the genes were normalized and analyzed using the 2^−ΔΔCT^ method [47]. The specific primer sequences used in the RT-PCR analysis are listed in Table 1. 

### 4.9. Animal Study Design and Surgical Procedures

The rabbits were anaesthetized via intramuscular injection with tiletamine-zolazepam at a dose of 15 mg/kg (Zoletil 50, Virbac, Carros Cedex, France). Before surgery, the dorsal area of the rabbit cranium was shaved and the surgical area was disinfected with beta-iodine and local anesthesia consisting of 1.8 ml of 2% lidocaine with 1:100,000 epinephrine. After midline skin incision, muscle dissection, and periosteal elevation, the calvarial bone was carefully exposed. Four symmetrical round 6 mm diameter bone defects were prepared in the calvaria using a trephine bure under the copious irrigation of sterile saline (Figure 7a) [48]. Four experimental modalities were randomly allocated to the 48 defects, as follows: (1) Empty control; (2) Bio-Oss^®^ (30 mg, S-size, 0.25 to 1 mm, Geistlich Pharma AG, Wolhusen, Switzerland); (3) Bio-Oss^®^ seeded with 1 × 10^6^ BMSCs; and (4) Bio-Oss^®^ seeded with 1 × 10^6^ DPSCs. BMSCs and DPSCs were seeded on Bio-Oss^®^ scaffolds and immediately implanted into the bone defects. After the operation, the muscle layer was closed with a bioresorbable suture (Vicryl 4.0, Ethicon, Somerville, NJ, USA.) and the skin layer was sutured with a 4–0 size nylon suture (Unik Surgical Sutures MFG Co, Taipei, Taiwan). Post-operatively, the animals received antibiotics (Baytril^®^, Bayer, Leverkusen, Germany) (5.0 mg/kg, SC, BID) and analgesics (Rimadyl^®^, Pfizer, New York, USA) (4.0 mg/kg, SC, BID) by subcutaneous injection for 3 days to prevent infection and control pain, respectively [49]. The animals had free access to food and water and were monitored daily for any complications or abnormal behaviors during the healing period.

### 4.10. Micro-Computed Tomography Measurements

After 3 and 6 weeks of healing, the rabbits were sacrificed and tissue blocks with bone grafting materials were harvested. After fixation in 10% neutral buffered formalin, the samples were processed and scanned with a micro-CT (Bruker Skyscan 1172, Bruker, Belgium) running at a voltage of 50 kV, an electric current of 100 mA, and a pixel resolution of 18 μm with a 0.5-mm aluminum filter. After scanning, three-dimensional (3D) image models were reconstructed using CTAN image analysis software (Bruker, Kontich, Belgium). The optimal threshold for discriminating between the bone and grafting materials was determined. Finally, the bone volume density (BV/TV %), i.e., the percentage of the bone volume (BV) to the total tissue volume (TV) within the VOI, was determined and expressed as mean ± SD. 

### 4.11. Histology and Histomorphometric Analysis

After the micro-CT measurements, tissue samples were prepared for histological analysis. The samples were decalcified in Plank-Rychlo’s solution (MUTO Pure Chemicals Co., Tokyo, Japan) for 5 days, dehydrated in graded ethanol baths, and then embedded in paraffin. The embedded samples were cut into 4-μm-thick sections using a microtome and stained with hematoxylin-eosin (HE; Sigma, St. Louis, MO) following standard protocols. Stained slide images were acquired using a standard light microscope (Leica DM500, Leica Microsystems, Wetzlar, Germany) equipped with a SPOT digital camera (Diagnostic Instruments, Inc., Sterling Heights, MI, USA). In performing the histomorphometric analysis, four sites were randomly selected for each slide and the new bone formation area of each region was calculated using Image-Pro Plus 6.0 software (Media Cybernetics, Silver Spring, MI, USA). 

### 4.12. Immunohistochemical Analysis

After dewaxing thoroughly with xylene and ethanol, the endogenous peroxidase activity was quenched with 3% hydrogen peroxide for 15 min. Antigen retrieval was then performed with citrate buffer at 80 °C and pH 6.5 for 10 min for immunohistochemistry detection. The tissue sections were reacted and incubated in a 1:100-diluted anti-collagen I antibody solution and a 1:100 diluted anti-osteoprotegerin (OPG) solution (Novus Biologicals, Littleton, CO, USA) at 37 °C for 1 h. The specimens were further incubated in a horseradish peroxidase-conjugated secondary antibody (Novus Biologicals, Littleton, CO, USA) for 30 min at room temperature. Finally, the sections were counterstained with hematoxylin after developing with 3,3′-diaminobenzidine tetrahydrochloride (DAB) (DAKO, Glostrup, Denmark), resulting in a brown color [50,51]. Histologic observations and images were acquired using a TissueFAXS plus system (TissueGnostics GmbH, Vienna, Austria) under ×10 magnification. The DAB total area / tissue total area (%) was determined using HistoQuest (TissueGnostics, Los Angeles, CA, USA) analysis software.

### 4.13. Statistical Analysis

All of the data were expressed as mean ± standard deviation (SD). The statistical analyses were performed using SPSS for Windows (Version 19, SPSS Inc., Chicago, IL, USA). Differences among the experimental groups were examined by one-way analysis of variance (ANOVA) tests using Tukey’s honest significant difference test. For all tests, a *p* value < 0.05 was considered to be statistically significant.

## 5. Conclusions

The results obtained in this study show that BMSCs and DPSCs combined with Bio-Oss xenografts provide equally excellent support in promoting bone regeneration in rabbit calvarial defects. This finding implies that DPSCs may offer an excellent source of MSCs for craniofacial bone regeneration. Overall, the in vitro and in vivo observations serve as a useful reference for future research on cell therapy.

## Figures and Tables

**Table 1:** Primers for RT-PCR analysis of osteogenic differentiated genes.

Primers Used for RT-PCR Analysis				
Gene	Type	Primers	Accession	Product Length
Runx2	Forward	5′-TCAGGCATGTCCCTCGGTAT-3′	XM_017345160	54
	Reverse	5′-TGGCAGGTAGGTATGGTAGTGG-3’		
ALP	Forward	5′-ACTGTGGACTACCTCTTG-3′	XM_017346489	76
	Reverse	5′-GGTCAGTGATGTTGTTCC-3′		
Osteocalcin	Forward	5′-ACTCTTGTCGCCCTGCTG-3′	XM_002715383	116
	Reverse	5′-CTGCCCTCCCTCTTGGAC-3′		
GAPDH	Forward	5′-GCCTGGAGAAAGCTGCTAAGT-3′	NM_001082253	133
	Reverse	5′– GAGTGGGTGGCACTGTTGAA-3′		
